# Upcycling Melon Peel Powder as a Value-Added Ingredient for Biscuits with Improved Nutritional and Antioxidant Properties

**DOI:** 10.3390/foods15142510

**Published:** 2026-07-15

**Authors:** Mariana-Atena Poiana, Daniela Stoin, Mariana Suba, Catalin Ianasi, Andreea Ghitulescu

**Affiliations:** 1 Faculty of Food Engineering, University of Life Sciences “King Mihai I” from Timisoara, Aradului Street No 119, 300645 Timisoara, Romania; marianapoiana@usvt.ro (M.-A.P.); andreea.ghitulescu@usvt.ro (A.G.); 2 “Coriolan Dragulescu” Institute of Chemistry, Romanian Academy, Bd. Mihai Viteazul No. 24, 300223 Timisoara, Romania; mariana_suba@acad-icht.tm.edu.ro (M.S.); ianasic@acad-icht.tm.edu.ro (C.I.); 3 Doctoral School Engineering of Plant and Animal Resources, University of Life Sciences “King Mihai I” from Timisoara, Aradului Street No. 119, 300645 Timisoara, Romania

**Keywords:** agro-industrial by-products valorization, melon peel powder, biscuits, phytochemicals, structural characterization, circular economy

## Abstract

In recent years, the food industry has increasingly focused on reintegrating nutrient- and phytochemical-rich processing by-products into high-consumption food matrixes. Aligned with this trend, our study investigated melon peel powder (MPP) as a value-added, upcycled ingredient in biscuit formulations with 0, 5, 10, 15, and 20% (*w*/*w*) MPP as a partial replacement for wheat flour (WF). The effects of this substitution on nutritional profile, colour, physical properties, total phenolic content (TPC), total flavonoid content (TFC), DPPH radical scavenging activity, ferric reducing antioxidant power (FRAP), structural characteristics, and post-baking retention were evaluated. MPP exhibited higher TPC (1531.76 mg GAE/100 g DM), TFC (681.42 mg QE/100 g DM), FRAP (104.37 µM Fe^2+^/g DM) and DPPH (125.01 µM TE/g DM) than WF. Its incorporation improved nutritional quality by increasing dietary fiber and ash, while reducing available carbohydrates and energy value. Substantial enhancements were observed, with up to 6.72- and 6.93-fold increases in FRAP and DPPH, alongside 3.82- and 3.92-fold increases in TPC and TFC at the highest substitution level. Although baking reduced these levels, retention remained at 57–62% (TPC), 55–59% (TFC), 60–65% (FRAP), and 63–70% (DPPH), indicating partial thermal stability and possible contribution of heat-induced antioxidant compounds. MPP addition also affected technological properties, increasing baking yield and spread ratio while decreasing lightness (L*) and increasing yellowness (b*) and browning index (BI), resulting in yellow-brown tones. Fourier-transform infrared spectroscopy (FTIR) and wide-angle X-ray scattering (WAXS) analyses confirmed structural compatibility between WF and MPP, evidenced by the absence of new functional groups, preservation of the A-type crystalline structure of wheat starch without polymorphic transitions, and no indication of phase separation, whereas baking promoted starch gelatinization and integration of fibrous and pectic components. These findings support a potential valorisation pathway for melon by-products through their conversion into a value-added ingredient for biscuit production.

## 1. Introduction

The increasing interest in food products with improved nutritional quality and potential health benefits has promoted the development of value-added formulations enriched with agro-industrial by-products [1,2]. Conventional bakery products are rich in refined carbohydrates and contain limited dietary fiber and bioactive compounds, reducing their nutritional value [3]. Furthermore, consumer interest in nutrient-dense and functional foods has driven the food industry to explore novel ingredients obtained from agro-industrial wastes as sources of valuable bioactive compounds [4]. Addressing this need, the partial replacement of wheat flour with fruit by-product-derived powders has emerged as a promising approach to create products with improved nutritional and bioactive profile [5]. Peel flours and powders from fruit processing by-products have attracted growing interest due to their high levels of dietary fiber, phenolics, minerals, and vitamins, which contribute to their antioxidant potential and associated bioactive properties [6]. Their valorisation promotes a circular economy [7,8,9], aligning with the broader shift toward nutritious and innovative food products [10].

Melon (*Cucumis melo* L.) by-products are especially interesting due to their large generation volumes and valuable compositional profile [2,3]. Peel and seed fractions represent a considerable proportion of the fruit mass, with the peel accounting for approximately 25–44% of the whole fruit, yet these materials remain largely underutilized and are commonly directed to animal feed or used for pectin and phytochemical recovery [7,11]. As inexpensive sources of bioactive compounds, melon by-products have considerable potential for the development of functional food ingredients [3,12]. Melon peel, in particular, is a rich source of phenolics, flavonoids, vitamins, and minerals. Compared with the edible pulp, melon peel contains higher levels of these bioactive compounds, contributing to its strong antioxidant properties [13,14,15]. Peel powders obtained from different melon varieties have been shown to exhibit high phenolic content and antioxidant activity [3,16]. In addition to its bioactive compounds, melon peel powder is also a valuable source of both soluble and insoluble dietary fiber, highlighting its potential as a fiber-rich food ingredient [2,8,9]. Its dietary fiber fraction consists of approximately 13% pectin, 20% cellulose, and 23% hemicellulose [17]. Soluble dietary fiber, particularly pectin, exhibits a high water-holding capacity and contributes to viscosity development, moisture retention, and texture modification. In contrast, insoluble dietary fiber, mainly cellulose and hemicellulose, provides structural support, increases water absorption, and may interfere with gluten network formation by competing for available water [9,18]. Therefore, the use of MPP as a food ingredient may influence the technological and quality characteristics of the resulting products.

The phytochemical profile and antioxidant activity of melon peel flour have been widely documented, highlighting its potential as a value-added ingredient for novel food development due to its considerable amount of dietary fiber and bioactive compounds with important antioxidant properties [19,20]. The application of melon by-products in bakery products has been investigated in several studies, summarized in Table 1.

Biscuits are considered suitable carriers for ingredients with potential functional properties due to their widespread consumption, convenience, portability, and formulation flexibility [23,24]. Consequently, they have attracted considerable interest as matrices for the creative utilization of fruit by-products in the development of value-added foods [3,7,24,25], particularly through the incorporation of fruit peel powders as partial wheat flour replacers. From a technological perspective, biscuits offer advantages for wheat flour substitution because of their simple processing, absence of fermentation, and limited gluten development requirements, allowing higher levels of ingredient incorporation than many other bakery products. The rationale of the present study is based on the high dietary fiber and bioactive compound content of melon peel powder, making it a valuable food ingredient while contributing to the upcycling of agro-industrial waste [1,2]. Most available studies have focused primarily on nutritional improvement and antioxidant activity, whereas information concerning structural organization within the biscuit matrix remains limited. Furthermore, moderate incorporation levels, typically around 10–15%, are generally considered suitable for maintaining product quality, while higher levels may adversely affect texture, colour, and overall acceptability [3,22,23,25,27]. Therefore, a better understanding of the structural changes associated with melon peel incorporation may provide complementary insight into the nutritional, technological, and antioxidant characteristics of enriched biscuit formulations.

To address this gap, the novelty of the present study stems from the systematic integration of Fourier-transform infrared spectroscopy (FTIR) and Wide-angle X-ray scattering (WAXS) structural characterization with conventional nutritional, physicochemical, and antioxidant property evaluations. FTIR was used to investigate molecular changes and potential interactions between wheat flour components and melon peel-derived polysaccharides, proteins, and dietary fiber, whereas WAXS was employed to assess changes in starch crystalline organization induced by partial substitution with melon peel powder. This integrated approach provides deeper insight into the structural modifications induced by melon peel powder incorporation and their implications for the physicochemical properties of biscuit matrices. In light of the aforementioned considerations, the primary objective of this study was to develop biscuit formulations by partially replacing wheat flour with melon peel powder (0–20%) and to evaluate the effects of MPP incorporation on their nutritional, physicochemical, antioxidant, and structural properties. A secondary objective was to investigate the retention of bioactive compounds after baking and to characterize the structural modifications revealed by FTIR and WAXS, thereby providing additional insight into the physicochemical properties of the final biscuits.

## 2. Materials and Methods

### 2.1. Chemicals and Analytical Reagents

Analytical-grade chemicals and reagents were used throughout the study. The Folin–Ciocalteu reagent, anhydrous sodium carbonate, sodium hydroxide, and ferric chloride hexahydrate were supplied by Merck (Darmstadt, Germany). Ethanol (96%), glacial acetic acid, and 0.1 M hydrochloric acid were provided by Merck (Darmstadt, Germany). Analytical standards, including gallic acid and quercetin, as well as 1,1-diphenyl-2-picrylhydrazyl (DPPH), 2,4,6-tris(2-pyridyl)-s-triazine (TPTZ), 6-hydroxy-2,5,7,8-tetramethylchroman-2-carboxylic acid (Trolox), ferrous sulfate heptahydrate, anhydrous sodium acetate, sodium nitrite, and aluminum nitrate nonahydrate, were sourced from Sigma-Aldrich (Taufkirchen, Germany).

### 2.2. Melon Peel Powder Preparation

Melon peel obtained from Canary melon (*Cucumis melo* var. *inodorus*), purchased from a supermarket in Romania, was used as raw material. First, the fruits were cleansed under flowing tap water, and then they were rinsed with distilled water to strip away surface impurities [19]. After washing, the fruits were gently dried with paper towels to eliminate residual moisture. The peel was removed from the fresh fruits using a stainless steel knife, sliced into small pieces and subjected to drying in a forced-convection hot air oven (Binder convective oven, Binder GmbH, Tuttlingen, Germany) at 55 °C for 24 h, until reaching a low moisture content (≈5%), a level considered adequate for microbial safety, following processing conditions reported for ensuring efficient dehydration while preserving bioactive compounds [33]. The dried material was cooled to 20 °C and ground with a Grindomix GM 200 cutting mill (Retsch GmbH, Haan, Germany), then passed through a 250 µm sieve to obtain a uniform and fine melon peel powder (MPP), followed by vacuum-sealing and preservation at 4 °C until further testing and preparation of the composite flours.

### 2.3. Preparation of Composite Flours and Biscuit Processing

The obtained dried melon peel powder (MPP) and type 000 wheat flour (WF), purchased from a local supermarket (Auchan, Timiș County, Romania), were used to prepare WF-MPP composite flours by substituting WF with MPP at percentages of 5%, 10%, 15%, and 20% by weight. The resulting blends, containing 95%WF–5%MPP, 90%WF–10%MPP, 85%WF–15%MPP, and 80%WF–20%MPP, were designated as 95WF5MPP, 90WF10MPP, 85WF15MPP, and 80WF20MPP, respectively. Preliminary laboratory-scale trials were conducted to establish the technological conditions for dough development and biscuit processing and to verify the applicability of the substitution levels selected based on the literature [3,23,28]. Four fortified biscuit formulations and one control formulation (100% WF) were developed using wheat flour or the previously prepared WF–MPP composite flours according to Table 2 and labelled as BWF, B95WF5MPP, B90WF10MPP, B85WF15MPP, and B80WF20MPP.

**Figure 1:**
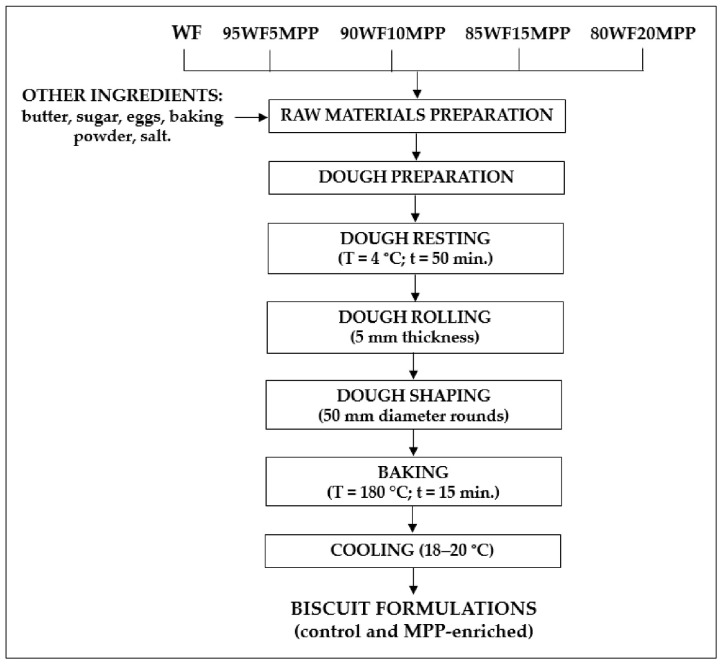
Process flow diagram of melon peel powder-enriched biscuit production. WF: wheat flour; MPP: melon peel powder; 95WF5MPP, 90WF10MPP, 85WF15MPP, 80WF20MPP: composite flours with partial replacement of WF by MPP at 0, 5, 10, 15, and 20% (*w*/*w*).

Experimental data were obtained from each formulation prepared in triplicate under identical conditions on the same day. As illustrated in Figure 1, the technological flowchart presents the main stages involved in the production of MPP-enriched biscuits.

The ingredients used in the formulations, including granulated sugar (Agrana Romania S.R.L., Buzău, Romania), butter (80% fat) (Covalact S.A., Sfântu Gheorghe, Romania), eggs (Regalina, Râșnov, Romania), baking powder (Fuchs Condimente RO S.R.L., Curtea de Argeș, Romania), and salt (Salrom S.A., Bucharest, Romania), were purchased from a local supermarket (Carrefour Hypermarket, Timisoara, Romania). Biscuit dough formulations were prepared by first mixing butter and sugar in a planetary mixer (Moulinex, 800 W, Moulinex SA, Paris, France) until a creamy consistency was obtained. Eggs were then added under continuous mixing, followed by baking powder and salt. Finally, previously sieved wheat flour or composite flour blends were gradually incorporated into the mixture. The resulting mass was further mixed for an additional 2 min until a smooth and homogeneous dough was formed [3,34]. The dough formulations, designated as DWF, D95WF5MPP, D90WF10MPP, D85WF15MPP, and D80WF20MPP, were subsequently kneaded manually for approximately 5 min on a clean surface and allowed to rest at 4 °C for 50 min to enable dough relaxation. After the resting period, the dough was rolled out using a rolling pin on a wooden board to a uniform thickness of approximately 5 mm. Circular dough pieces were cut using a stainless-steel cutter with a diameter of 5 cm, transferred to lightly greased baking trays, and baked in a preheated convection oven (FM RXB 610 V7, FM Industrial S.A., Lucena, Spain) at 180 °C for 15 min. Dough samples from each formulation were collected, packed in polypropylene bags, and stored at −20 °C until chemical analysis. After baking, biscuits were cooled to room temperature. Biscuit samples from each batch were randomly selected, sealed in polypropylene bags, and stored at −20 °C under the same conditions as dough samples until analysis. In parallel, a separate set of biscuits from each batch was used for the determination of physical characteristics after cooling at room temperature for 3 h.

### 2.4. Proximate Analysis and Energetic Value of Wheat Flour, Melon Peel Powder, and Biscuits

The proximate analysis of the raw materials (WF and MPP) alongside the enriched biscuits was evaluated according to AOAC Official Methods [35] by determining moisture (925.10), fat (922.06), protein (920.87), total dietary fiber (991.43), and ash content (923.03). The available carbohydrate content was calculated by difference as the remaining percentage after subtracting the measured contents of moisture, fat, protein, total dietary fiber, and ash from 100 [36]. The energy value (kcal/100 g) was estimated by summing the caloric contributions of macronutrients by applying conversion factors of 4 kcal/g for both carbohydrates and proteins, and 9 kcal/g for fats [37].

### 2.5. Color Parameters Analysis

The color attributes of the samples were evaluated using a CR-5 Benchtop Colorimeter (Konica Minolta, Sakai, Osaka, Japan) operating in the CIELAB color space. The measured parameters included color coordinates $L^{*}$, $a^{*}$, and $b^{*}$, representing lightness, red–green, and yellow–blue axes, respectively [38]. In the CIELAB color space, redness and greenness are denoted by positive and negative $a^{*}$ values, whereas yellowness and blueness are represented by positive and negative $b^{*}$ coordinates, respectively [38]. Based on the recorded $L^{*}$, $a^{*}$, and $b^{*}$ values, the total color intensity (TCI) was quantified in accordance with Equation (1) [25].

(1) $$TCI=\sqrt{{\left(L^{*}\right)}^{2}+{\left(a^{*}\right)}^{2}+{\left(b^{*}\right)}^{2}}$$

Chroma (C), representing color saturation or purity, was determined by applying the formula described in Equation (2) [39]:

(2) $$C=\sqrt{{\left(a^{*}\right)}^{2}+{\left(b^{*}\right)}^{2}}$$

The hue angle ($h^{{}^{\circ}}$), describing the dominant color tone, was quantified in accordance with Equation (3) [39]:

(3) $$h^{{}^{\circ}}=tan^{-1}\left(\frac{b^{*}}{a^{*}}\right)$$

The overall color difference ∆E was determined by comparing each MPP-enriched biscuit sample with the control using the standard CIE equation (Equation (4)) taking into account the corresponding value recorded for $L^{*}$, $a^{*}$, and $b^{*}$ [39]:

(4) $$\Delta E=\sqrt{{\left(L^{*}-L_{0}^{*}\right)}^{2}+{\left(a^{*}-a_{0}^{*}\right)}^{2}+{\left(b^{*}-b_{0}^{*}\right)}^{2}}$$

where $L^{*}$ and $L_{0}^{*}$ represent the lightness of the sample with MPP addition and control sample, respectively; $a^{*}$ and $a_{0}^{*}$ represent the red–green values of the sample with MPP addition and control sample, respectively; $b^{*}$ and $b_{0}^{*}$ represent the yellow–blue values of the sample with MPP addition and control sample, respectively.

The browning index (BI) was calculated from the color coordinates to assess the degree of color change at the surface of the biscuit samples following the incorporation of MPP, by applying the relationships outlined in Equations (5) and (6) [38]:

(5) $$BI=\frac{\left(X-0.31\right)\cdot 100}{0.17}$$

(6) $$X=\frac{a^{*}+1.75\cdot L^{*}}{5.645\cdot L^{*}+a^{*}-3.012\cdot b^{*}}$$

### 2.6. Assessment of Baking Performance and Physical Characteristics of Formulated Biscuits

Following the baking process, the mass of the resulting biscuits for every formulation was quantified, alongside the baking loss calculated relative to the initial dough mass. Baking yield was subsequently calculated as the ratio of baked biscuit weight to initial dough weight [40]. The diameter, thickness, and spread ratio of the biscuits after cooling to room temperature were determined [41]. For diameter measurement, six randomly selected biscuits were aligned edge to edge and measured using a digital caliper; the arrangement was then rotated by 90° and remeasured, and the mean diameter was calculated by averaging the two values and dividing by six. Biscuit thickness was determined by stacking six biscuits vertically and measuring their combined height in different orientations using the same caliper, with the mean value used for calculation. The spread ratio was expressed as the ratio of mean diameter to mean thickness, and the spread factor was calculated relative to the control as the spread ratio multiplied by 100 [42].

### 2.7. Evaluation of Phytochemical Content, DPPH Radical Scavenging Activity and Ferric Reducing Antioxidant Power (FRAP) of Wheat Flour, Melon Peel Powder, Dough, and Biscuits

#### 2.7.1. Ethanolic Extraction Procedure for Sample Preparation

Ethanolic extracts were prepared from WF, MPP, dough, and biscuit samples according to Litwinek et al. [43], using 70% (*v*/*v*) ethanol and a two-step successive extraction procedure to ensure efficient recovery of bioactive compounds. The resulting extracts were used for the evaluation of total phenolic content, total flavonoid content and for assessing radical scavenging capacity and ferric reducing antioxidant potential. For each material, approximately 1.0 g was dispersed in 10 mL of 70% (*v*/*v*) ethanol and subjected to continuous shaking for 2 h at room temperature using the linear shaker SK-L330-Pro (Ningbo Scientz Biotechnology Co., Ltd., Ningbo, China). Centrifugation of the resulting mixture was carried out at 10,000 rpm (9.5 cm rotor radius) for 10 min in a benchtop centrifuge model EBA 21 (Hettich, Tuttlingen, Germany). The solid residue was re-extracted with the same solvent, stirred for an additional hour, and centrifuged again under identical conditions. Both supernatants were merged and kept frozen (−20 °C) in complete darkness until subsequent testing. Samples were extracted in triplicate, yielding independent replicates for all further analytical determinations.

#### 2.7.2. Total Flavonoid Content Analysis

Total flavonoid content (TFC) was determined using a modified procedure adapted from Al-Farsi et al. [44]. Briefly, 3.0 mL of the ethanolic extract was mixed with 4.5 mL of distilled water and 1.0 mL of 0.3% sodium nitrite (NaNO_2_). After incubation at 20 °C for 6 min, 1.0 mL of 10% aluminum nitrate [Al(NO_3_)_3_] was added and the mixture was allowed to react for another 6 min. Following this, alkaline conditions were established by adding 10 mL of 4% (*w*/*w*) NaOH, with the total volume being adjusted to 25 mL with 70% ethanol. After allowing the reaction to proceed for 15 min, the optical density was read at 510 nm on a Specord 205 UV–Vis spectrophotometer (Analytik Jena AG, Jena, Germany), using 70% ethanol as the reference blank. Results were reported as quercetin equivalents (QE) per 100 g dry matter (DM) after determining concentrations from a quercetin calibration curve (0.5–50 µg/mL).

#### 2.7.3. Total Phenolic Content Analysis

Following the protocol reported by Gómez-García et al. [45], the total phenolic content (TPC) was quantified through the Folin–Ciocalteu colorimetric method. The ethanolic extracts were analyzed directly, except for the MPP sample, which was diluted 1:10 (*v*/*v*) with 70% ethanol prior to analysis. To perform the quantification, 2.5 mL of Folin–Ciocalteu reagent (diluted 1:10 with distilled water) were added to 0.5 mL of each extract, followed by the addition of 2 mL of 7.5% Na_2_CO_3_ solution. The reaction mixture was incubated at 50 °C for 30 min in a Memmert INB500 incubator (Memmert, Schwabach, Germany), after which the absorbance was determined at 750 nm. A reagent blank prepared under identical conditions was used as reference. Results for TPC were calculated using a gallic acid calibration curve (0.1–1.0 µmol/mL) and reported in terms of mg gallic acid equivalents (GAE)/100 g DM.

#### 2.7.4. Free Radical Scavenging Activity via 1,1-Diphenyl-2-picrylhydrazyl (DPPH) Assay

Following the methodology of Dimov et al. [46], the free radical scavenging capacity of the samples was evaluated. The assay relies on the reduction of stable DPPH radicals into DPPH-H through hydrogen or electron donation from the antioxidant extracts. A 0.1 mM DPPH reagent was prepared using 70% ethanol as the solvent. Undiluted extracts from WF, dough, and biscuit samples were used in the measurements, while the MPP extract was diluted 1:10 (*v*/*v*) with 70% ethanol prior to analysis. For each determination, 1.0 mL of the appropriately diluted extract was combined with 2.5 mL of the DPPH solution. The mixtures were homogenized and, following a 30 min incubation period in darkness at 20 °C, the spectrophotometric response was recorded at 517 nm against a 70% ethanol blank. A control sample containing only 70% ethanol and the DPPH solution was processed in parallel under identical conditions. The decrease in absorbance relative to the control was used to estimate the percentage of DPPH radical scavenging. A calibration curve was established by plotting the DPPH radical scavenging percentage against the corresponding concentrations of Trolox standards (1.0–25 µg/mL). The DPPH radical scavenging capacity of the samples was calculated via interpolation from the reference curve, with results expressed as µmol Trolox equivalents (TE)/g DM [47].

#### 2.7.5. Ferric-Reducing Antioxidant Power (FRAP) Assay

The FRAP assay was employed to evaluate the ferric reducing antioxidant power of the samples, monitoring the ability of the extract compounds to convert the Fe^3+^-TPTZ complex into its colorful Fe^2+^-form under acidic conditions. This mechanism directly reflects the electron-donating and chemical reducing capacity of the tested samples, generating a blue-colored complex that exhibits a strong absorption maximum at 593 nm [48]. The FRAP working reagent was freshly prepared by combining 100 mL of acetate buffer (pH 3.6) with 10 mL of a 10 mM TPTZ solution in 40 mM HCl and 10 mL of a 20 mM FeCl_3_·6H_2_O solution. While WF and biscuit extracts were analyzed directly, MPP and dough extracts required prior dilutions with 70% (*v*/*v*) ethanol at 1:50 and 1:2 (*v*/*v*), respectively. In each analysis, a 0.5 mL aliquot of extract was blended with 2.5 mL of FRAP reagent and kept at 37 °C for a 30 min incubation period. Spectrophotometric measurements were done at 593 nm relative to a blank. The ferric reducing antioxidant power was computed via a ferrous sulfate standard curve (0.05 to 0.5 µmol Fe^2+^/mL) with final values reported as µM Fe^2+^/g DM.

### 2.8. FTIR-ATR Characterization of WF, MPP, Composite Flours, and Biscuit Samples

Fourier Transform Infrared Spectroscopy with Attenuated Total Reflectance (FTIR-ATR) was used to identify functional groups and structural changes in wheat flour (WF), melon peel powder (MPP), WF–MPP composite flours, and formulated biscuits. A Nicolet™ iS50 FTIR spectrometer (Thermo Fisher Scientific, Waltham, MA, USA) configured with a diamond ATR accessory was utilized to collect the FTIR spectra of the sample at ambient temperature. Samples were subjected to an overnight drying step at 40 °C to ensure the removal of residual moisture that could interfere with spectroscopic measurements. This low-temperature drying procedure was applied to prevent possible structural or thermal modifications of the samples. Post drying, samples were placed immediately on the ATR crystal setup. Spectra were then generated from 4000 to 400 cm^−1^ using a resolution of 4 cm^−1^ and recording 32 scans for each measurement [36].

### 2.9. Wide-Angle X-Ray Scattering (WAXS) of WF, MPP, Composite Flours and Biscuit Samples

Wide-angle X-ray scattering (WAXS) was used to characterize the crystalline structure of wheat flour (WF), melon peel powder (MPP), WF–MPP composite flours, and biscuit formulations. Briefly, a Xenocs Xeuss 3.0 set-up (Xenocs SAS, Grenoble, France), outfitted with a Cu Genix 3D X-ray source and a Dectris Eiger2 Si 1M detector (DECTRIS Ltd., Baden-Daettwil, Switzerland), served for performing all measurements. Data collection was performed at room temperature under vacuum using a Kapton film support, a 45 mm specimen-to-detector distance, H-Flux collimation, and 300 s of exposure [36]. Subsequent the collected data were processed via XSACT software (v. 2.10; Xenocs, Grenoble, France) [49].

### 2.10. Statistical Analysis

Results are expressed as mean ± standard deviation (SD) of three independent batches (*n* = 3). Differences among wheat flour and melon peel powder samples, dough formulations, and biscuits were evaluated by one-way ANOVA followed by Tukey’s post hoc test. Homogeneity of variances was assessed using Levene’s test, and normality was examined prior to analysis. Statistical significance was set at *p* < 0.05.

## 3. Results and Discussion

### 3.1. Proximate Analysis of Wheat Flour, Melon Peel Powder, and Biscuits

The proximate composition and energy value of wheat flour (WF) and melon peel powder (MPP) are presented in Table 3, highlighting the compositional differences between the two raw materials.

MPP showed lower moisture, protein, carbohydrate content, and energy value, along with higher ash, fat, and total dietary fiber contents compared to WF, indicating its potential as a functional ingredient for nutritionally enhanced bakery products. The moisture content of MPP (4.97%) was much lower than that of WF (12.89%), which may contribute to improved storage stability and reduced microbial spoilage. Similar trends have been reported in melon peel powders, highlighting the importance of low moisture levels for extending the shelf life of fruit by-products [24,28]. The value obtained was lower than those previously reported (12.13%) [26] and (8.71%) [24], which may be attributed to differences in drying conditions and processing methods. The protein content of MPP (10.09%) was lower than that of WF (11.48%), reflecting the lower protein contribution of fruit by-products compared with cereal flours. This value falls within the range reported for MPP (8.41–10.10%) [9] and is close to that reported for sharlyn melon peel (9.07%) [25], confirming consistency with similar raw materials. Higher values of (12.28%) [28] and (12.97%) [8], as well as lower values of (7.53%) [26] and (6.91%) [24], have also been reported. These differences are mainly associated with cultivar, maturity stage, agronomic conditions, and processing methods.

The lipid content of MPP (1.66%) was slightly higher than that of WF (1.28%), consistent with the generally low-fat nature of both materials. Similar values have been reported for MPP, including sharlyn melon peel (1.58%) [25], (1.42%) [8], and (1.74%) [28], supporting the stability of low lipid levels in melon by-products.

The ash content of MPP (9.71%) was markedly higher than that of WF (0.34%), consistent with a higher mineral fraction in melon peel powder. Previous studies have reported comparable values, such as (10.2%) [24] and (10.48%) [8], confirming the high mineral content typical of melon peel flours. Lower values were also reported (8.36%) [3] and (5.89%) [26], possibly due to varietal and agronomic differences.

The total dietary fiber content of MPP (28.39%) was much higher than that of WF (0.46%), confirming its role as a fiber-rich ingredient. This value falls within the range reported in previous studies, including higher values (50%) [24] and (47.1%) [9], comparable values (36.76%) [26] and (29.59%) [25], and lower values (18%) [8], (17.39%) [28], and (12.06%) [3], reflecting differences among melon varieties and processing conditions.

The available carbohydrate content of MPP (45.18%) was lower than that of WF (73.55%), mainly due to its higher fiber and ash fractions. Reported values vary widely, from around 23.70% [24] to 57.13% [8], depending on cultivar, maturity stage, and agroclimatic conditions. The energy value of MPP (236.02 kcal/100 g) was lower than that of WF (351.64 kcal/100 g), reflecting differences in macronutrient composition, particularly lower available carbohydrates and slight variations in protein and lipid contents.

The proximate composition and energy value of MPP-enriched biscuit formulations are summarized in Table 4, highlighting the contribution of melon peel powder incorporation to the nutritional profile.

The partial replacement of WF with MPP significantly influenced (*p* < 0.05) the proximate composition and energy value of biscuit formulations, except for protein content, demonstrating the nutritional impact of increasing substitution levels in the final baked product matrix. Moisture content increased significantly (*p* < 0.05) with increasing MPP incorporation, from 5.08% in the control (BWF) to 6.33% in B80WF20MPP. This parameter is important in biscuit formulations, as it influences texture, crispness, and storage stability [3]. A similar trend was reported by Hussain et al. [3], where moisture increased from 8.15% to 10.11% in biscuits fortified with melon peel powder, indicating consistent effects of MPP on water distribution in bakery systems. Comparable increases were also observed by dos Santos et al. [28] in MPP-enriched muffins (29.14% to 35.61%), confirming the moisture retention capacity of fiber-rich by-products. This behaviour is attributed to the high water-binding capacity of dietary fiber in melon peel powders, which enhances water retention and increases bound water within the matrix. Similar effects were reported in MPP-fortified cookies (4.16–5.51%) [32] and in the MPP-enriched bread [26]. Eshak [50] also associated moisture variation in fiber-enriched bakery products with the higher water-holding capacity of fruit peel materials compared to wheat flour. Despite this trend, moisture levels remained within the typical range for biscuits.

Protein content showed a slight decreasing trend with increasing MPP incorporation, from 8.07% in the control formulation (BWF) to 7.96% in B80WF20MPP, with no significant differences (*p* > 0.05) among samples. This reduction is attributed to the lower protein content of MPP compared to WF. Similar decreases have been reported in biscuits fortified with watermelon and muskmelon peel powders [3], MPP-enriched cookies [32], MPP-supplemented bread [26], and cakes containing watermelon rind and sharlyn melon peel powder [25], all linked to the lower protein content of the fruit peel-based ingredients.

Ash content increased significantly (*p* < 0.05) with increasing MPP incorporation, from 1.03% in the control (BWF) to 2.01% in B80WF20MPP. A similar trend has been reported in MPP-enriched biscuits, where higher ash values were associated with muskmelon peel addition due to its higher mineral content compared to wheat flour [3]. Comparable increases were observed in MPP-enriched cookies (≈25% increase) [32], bread formulations with progressive ash increases at higher substitution levels [26], cakes containing watermelon rind and sharlyn melon peel powders (1.48% to 2.04–2.11%) [25], and muffins enriched with melon peel flour (0.891% to 1.533%) [28], confirming the mineral-rich nature of MPP.

Fat content showed a slight but significant (*p* < 0.05) increasing trend with increasing MPP incorporation, from 23.29% in the control (BWF) to 23.56% in B80WF20MPP. A similar variation was reported by Hussain et al. [3] in MPP-fortified biscuits, where fat content ranged from 23.95% in the control to 24.88% at higher incorporation levels, with minimal changes attributed to the low lipid contribution of MPP. Comparable findings were also reported in cakes enriched with melon peel powder and watermelon rind flour [25], where fat content showed only minor variations with increasing substitution levels.

Dietary fiber is an important parameter in food formulation, as a high dietary fiber content supports the use of ingredients as functional components and is associated with various health benefits [8]. Accordingly, its content represents a key compositional indicator in bakery products, reflecting the contribution of fiber-rich ingredients to the nutritional profile of the final product. Total dietary fiber content increased significantly (*p* < 0.05) with increasing MPP levels, from 0.25% in the control (BWF) to 3.19% in B80WF20MPP. A similar trend was reported in MPP-fortified biscuits, where total dietary fiber increased from 0.52% in the control to 4.82% (canary melon), 3.62% (watermelon), and 6.69% (muskmelon) [3]. Comparable increases were observed in MPP-enriched muffins (1.71% to 3.88–4.45%) [28], while higher substitution levels led to a more pronounced increase in fiber content in biscuits containing 50% MPP (1.53% to 14.7%) [24], confirming the fiber-rich nature of melon peel ingredients relative to wheat flour.

The available carbohydrate content showed a significant decreasing trend (*p* < 0.05) with increasing MPP incorporation, from 62.28% in the control (BWF) to 56.93% in B80WF20MPP. A similar reduction in available carbohydrates has been reported in muffins and biscuits enriched with fruit peel powders, decreasing from 66.05% to 54.85% at higher substitution levels (20%) [28], and from 63.6% to 47.0% in MPP-containing biscuits [24], attributed to the replacement of wheat flour with fiber-rich ingredients. A gradual decrease was also observed in bread formulations (50.44% to 46.45%) at 12% MPP incorporation [26], confirming the consistent effect of melon peel by-products on lowering available carbohydrate content in bakery systems. The energy value of the biscuit samples decreased significantly (*p* < 0.05) with increasing MPP substitution, from 491.01 kcal/100 g in the control to 471.68 kcal/100 g in the sample with 20% MPP (B80WF20MPP). This reduction is associated with the higher dietary fiber content and the lower caloric contribution of MPP compared with WF. A similar decrease in energy value has been reported in MPP-fortified bakery products [24,28], attributed to the replacement of energy dense flour with fiber-rich ingredients, which reduces overall caloric density. Ertaş and Aslan [32] also observed a reduction in cookies (482.87 to 475.58 kcal) at 5% MPP, supporting the same trend.

### 3.2. Color Parameters of Biscuit Formulations

**Figure 2:**
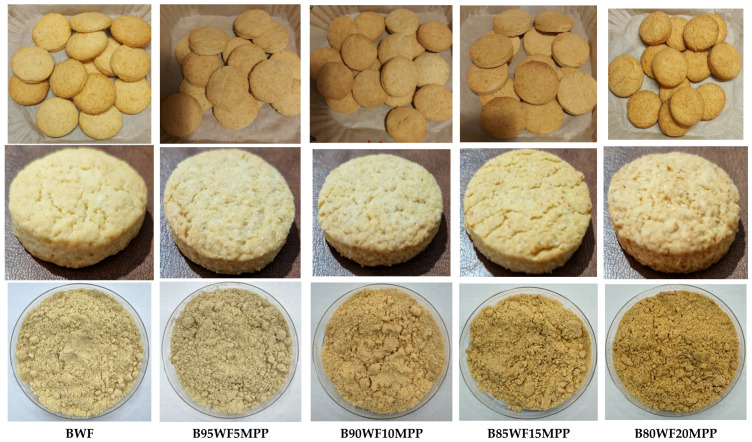
Biscuits enriched with melon peel powder. BWF, B95WF5MPP, B90WF10MPP, B85WF15MPP, B80WF20MPP: biscuits with partial replacement of WF by MPP at 0, 5, 10, 15, and 20% (*w*/*w*).

Figure 2 illustrates the biscuit formulations prepared from WF-MPP composite flours.

Table 5 presents the color parameters of biscuit formulations enriched with melon peel powder (MPP) in comparison with the control sample.

The incorporation of MPP significantly (*p* < 0.05) affected the color characteristics of the biscuits. As the substitution level increased, the lightness parameter ($L^{*}$) decreased from 79.97 in the control sample (BWF) to 74.85 in B80WF20MPP, with a dose-dependent reduction already evident at 5% MPP (79.15). This progressive decrease in $L^{*}$ indicates that even low levels of MPP affected biscuit appearance, with a more pronounced effect at higher substitution levels. The reduction in lightness reflects the development of darker tones in the final product, associated with Maillard reactions between reducing sugars and amino compounds during baking, leading to the formation of melanoidin pigments [51]. A concentration-dependent decrease in $L^{*}$ values with increasing MPP level has also been reported previously [19,51], as well as in MPP-enriched and other bakery products fortified with fruit by-product powders [25,32].

The redness parameter ($a^{*}$) increased from 4.21 in the control to 4.60 at 5% MPP and 5.89 at 20% MPP, indicating a gradual increase in red tonalities. The yellowness parameter ($b^{*}$) also increased significantly from 26.08 in the control to 27.60 at 5% and 31.11 at 20% MPP, showing a progressive intensification of yellow coloration. These changes are attributed to the natural carotenoid pigments present in MPP and its inherent yellowish color, which contribute directly to the observed increase in yellowness. An increase in $b^{*}$ values has been reported in melon by-product-enriched bakery products, attributed to their pigment composition [25,28].

The total color intensity (TCI) decreased slightly from 84.22 in BWF to 81.27 in B80WF20MPP. The hue angle ($h^{{}^{\circ}}$) showed a slight decrease from 80.83 in the control to 80.54 at 5% MPP and 79.28 at 20% MPP, indicating a shift toward more yellow hues. This observation is consistent with the increase in $b^{*}$ values and further supports the influence of MPP pigments on biscuit color. Similar results have been reported in cookies with 5% MPP, where a decrease in $h^{{}^{\circ}}$ from 87.74 in the control to 86.89 was observed [32].

The total color difference (ΔE), calculated relative to the control sample, showed statistically significant differences (*p* < 0.05) among all formulations, increasing progressively from 1.77 in B95WF5MPP to 7.37 in B80WF20MPP with increasing MPP levels. The obtained ΔE values were interpreted guided by the thresholds established by Mokrzycki and Tatol [52], which describe how a standard observer perceives color differences. According to these criteria: 0 < ∆E < 1—the color difference remains imperceptible to the observer; 1 < ∆E < 2—the difference is detectable exclusively by a trained evaluator; 2 < ∆E < 3.5—the difference becomes apparent to an untrained observer; 3.5 < ∆E < 5—a clear and evident color difference is easily perceived; 5 < ∆E—two entirely distinct colors are distinguished by the observer. Comparing the experimental data with these thresholds, the formulation with 5% MPP (∆E = 1.77) remains within the zone where the color differences are detectable exclusively by an experienced observer, meaning modifications are practically unnoticeable to untrained consumers. However, the formulation containing 10% MPP (∆E = 3.99) crosses into the range where the color difference is detectable to an unexperienced observer (∆E < 3.5) and is easily perceived as an evident difference (3.5 < ∆E < 5). Furthermore, ∆E values for substitution levels of 15% and 20% (∆0 = 5.70; 7.37) exceed the critical threshold of 5.0, where two completely distinct colors are distinguished by the observer. A total color difference greater than 5.0 marks the point where visual changes can change consumer expectations [38,52]. Since the 15% and 20% MPP levels exceeded this limit, these biscuits cannot be considered visually similar to the control baseline, highlighting a significant change in product appearance.

The browning index (BI) increased markedly from 42.52 in the control sample to 46.24 at 5% MPP and 58.19 at 20% MPP, indicating greater browning development in enriched biscuits. This increase may be associated with Maillard reactions and caramelization occurring during baking, as well as with the composition of MPP, as previously reported in biscuit systems enriched with alternative flours [34,38]. The formation of melanoidins during these reactions has been widely linked to the development of darker tones in baked products [53]. The combined effects of naturally occurring pigments and thermally induced browning reactions contributed to the progressive shift from a lighter color toward a more pronounced yellow-brown appearance as the level of MPP increased.

### 3.3. Physical Characteristics of Biscuit Formulations

Table 6 presents the effect of replacing WF with MPP on biscuit weight after baking, baking yield, and physical characteristics.

As shown in Table 6, the biscuits weight produced from 422 g of dough increased significantly (*p* < 0.05), from 362.78 g in the control sample (BWF) to 367.62 g in B80WF20MPP. Accordingly, baking weight loss decreased from 59.22 g to 54.38 g, indicating improved moisture retention with increasing MPP levels. This trend is consistent with previous results reported by Pawde et al. [54]. Consequently, baking yield increased from 85.97% in BWF to 87.11% in B80WF20MPP. Based on biscuit weight and a 200 g flour input per formulation, the flour required to produce 100 g of biscuits decreased progressively from 55.13 g in the control to 54.40 g in B80WF20MPP, with intermediate values of 54.94, 54.78, and 54.61 g at 5%, 10%, and 15% MPP, respectively.

Incorporation of MPP led to a significant, dose-dependent reduction in biscuit diameter, from 58.78 mm in the control sample to 56.12 mm at the 20% MPP level, accompanied by a more pronounced decrease in thickness, from 11.07 mm to 9.56 mm. As thickness decreased more than diameter, the spread ratio increased progressively from 5.31 to 5.87, while the spread factor reached 110.54% relative to the control at the highest substitution level. These results suggest that both the progressive dilution of the gluten network resulting from wheat flour replacement, which reduces the ability of the dough to maintain structure and height during baking, and the hydration properties of dietary fiber contributed to the observed changes in biscuit geometry [55,56].

As reported by Silva et al. [24], melon peel flour has a high water absorption capacity, which is related to the hydration properties of its dietary fiber, allowing MPP to retain a greater amount of water during dough formation. Consequently, less free water may be available for gluten development and starch gelatinisation, which may alter dough structure and reduce gas retention during baking, thereby contributing to the greater reduction in biscuit thickness compared to diameter. The same authors also noted that high substitution levels of wheat flour with melon peel flour may be challenging because its high water absorption capacity makes it more difficult to obtain a cohesive and uniform dough.

Ajila et al. [55] reported that incorporation of mango peel powder, a rich source of dietary fiber containing pectin and other polysaccharides with high water-holding capacity, increased water absorption and dough development time while reducing dough stability in wheat flour blends. The increase in water absorption was attributed to the high water-holding capacity of the dietary fiber, particularly pectin and other polysaccharides, whereas the reduction in dough stability was associated with dilution of the gluten network following partial replacement of wheat flour [55]. Similarly, Pawde et al. [54] reported that the high water absorption capacity of dragon fruit powder, resulting from its hydrophilic dietary fiber, leads to reduced dough fluidity and consequently limited biscuit diameter during baking. These findings are consistent with the progressive reduction in biscuit diameter observed in this study as the level of MPP increased. It has been documented that fruit powders and fruit by-products used in biscuit formulations significantly affect product geometry, depending on their dietary fiber content and hydration properties [57]. These insights indicate that the observed changes in biscuit geometry result from the combined effects of the hydration properties of MPP dietary fiber and gluten dilution caused by the partial replacement of wheat flour. Although the dimensional attributes were altered, the results support the use of MPP in fiber-enriched biscuit formulations.

### 3.4. Phytochemical Content, DPPH Radical Scavenging Activity, and Ferric Reducing Antioxidant Power of WF and MPP

Table 7 presents the total phenolic content (TPC), total flavonoid content (TFC), DPPH radical scavenging activity, and ferric reducing antioxidant power (FRAP) of WF and MPP.

As shown in Table 7, MPP exhibited markedly higher TPC, TFC, DPPH radical scavenging activity, and ferric reducing antioxidant power (FRAP) than WF, demonstrating its high antioxidant potential. The TPC of MPP reached 1531.76 mg GAE/100 g DM, compared with 99.41 mg GAE/100 g DM in WF. A similar pattern was observed for TFC, which was significantly higher in MPP (681.42 mg QE/100 g DM) than in WF (41.94 mg QE/100 g DM). These results indicate that melon peel is a rich source of phenolic compounds and flavonoids with recognized antioxidant properties. The TPC value obtained for MPP is consistent with previously reported data for melon by-products. Phenolic contents of 1976 and 2212 mg GAE/100 g DM have been reported in yellow and green melon peels, respectively, with phenolic acids, flavones, phenyl ethanoids, phenolic alcohols, hydroxybenzoic acids, and flavonoids identified as the main classes [24]. Another study also reported a TPC of 1709.6 mg GAE/100 g for MPP [24]. TPC ranging from 670 to 1035 mg GAE/100 g have been reported in cantaloupe peel extracts [15], while values up to 25.48 mg GAE/g have been observed in peel extracts from certain cultivars [58]. These variations may be attributed to differences in cultivar, maturity stage, processing conditions, and extraction methods.

The TFC value obtained for MPP falls within the range reported in the literature. Flavonoid contents between 3.25 and 7.02 µg CE/mg (equivalent to 325–702 mg CE/100 g) have been reported in cantaloupe peel extracts [15], which are comparable to those obtained in the present study. The flavonoid fraction may contribute to the antioxidant characteristics of MPP due to the radical scavenging activity of these compounds [15].

The ferric reducing antioxidant power of MPP reached 104.37 µM Fe^2+^/g DM, whereas WF exhibited only 2.74 µM Fe^2+^/g DM. Similarly, DPPH radical scavenging activity was significantly higher in MPP (125.01 µM TE/g DM) compared with WF (3.28 µM TE/g DM). These results highlight the strong antioxidant potential of MPP and are consistent with previous reports on melon by-products [58]. FRAP values ranging from 3.54 to 441.83 µmol Fe^2+^/g DM have been reported in processed tropical fruit peels [59], while higher antioxidant activity in melon peels compared with seeds has also been associated with greater polyphenol content [58].

The relatively low DPPH radical scavenging activity and ferric reducing antioxidant power of WF were consistent with values reported for refined wheat flours [60] and were supported by the corresponding TPC and TFC values reported in the literature [37,61,62].

TPC values of approximately 98–102 mg GAE/100 g DM have been reported in wheat flours [60,61], while DPPH activity ranging from 4.59 to 5.00 µmol TE/g has also been observed [62]. Flavonoid contents of around 50.12 mg QE/100 g have been reported in flour samples [37]. These variations among studies may be attributed to differences in wheat cultivar, milling degree, storage conditions, and extraction procedures.

The differences between WF and MPP (*p* < 0.05) confirm the higher antioxidant potential of melon peel powder and support its potential use as a functional ingredient for the enrichment of cereal-based products with bioactive compounds.

### 3.5. Phytochemical Content, DPPH Radical Scavenging Activity, and Ferric Reducing Antioxidant Power of Dough and Biscuit Formulations

The total phenolic content, total flavonoid content, DPPH radical scavenging activity, and ferric reducing antioxidant power (FRAP) of the MPP-supplemented dough formulations were determined before baking and compared to the control dough (Table 8).

MPP was used as a partial substitute for WF in the biscuit dough, while the remaining ingredients (sugar, fat, eggs, baking powder, and salt) were kept constant across formulations. Therefore, differences in investigated phytochemical profile can be mainly attributed to the increasing level of MPP in the flour fraction.

A progressive increase in TPC, TFC, DPPH radical scavenging activity, and ferric reducing antioxidant power (FRAP) was observed with increasing levels of MPP incorporation relative to the control dough (DWF). FRAP values increased with the level of substitution. The control formulation showed a FRAP value of 1.78 µM Fe^2+^/g DM, which increased to 4.04 µM Fe^2+^/g DM at 5% MPP and reached 11.97 µM Fe^2+^/g DM at 20% MPP, corresponding to an approximately 6.72-fold increase relative to the control. This improvement reflects the higher electron-donating capacity of antioxidant compounds introduced through MPP addition. The DPPH radical scavenging activity showed a similar increasing trend with MPP addition. The control dough exhibited a relatively low DPPH radical scavenging activity of 2.14 µM TE/g DM, whereas at 20% substitution the value reached 14.83 µM TE/g DM, corresponding to an approximately 6.93-fold increase compared with the control. This improvement indicates enhanced radical inhibition capacity associated with MPP. The parallel increase in FRAP and DPPH radical scavenging activity suggests that phenolic and flavonoid compounds introduced through MPP contributed to the antioxidant potential of the dough system.

TPC increased with MPP level, from 51.71 mg GAE/100 g DM in the control dough to 83.55 mg GAE/100 g DM at 5% substitution and 181.84 mg GAE/100 g DM at 20% substitution, corresponding to an approximately 3.52-fold increase. This confirms that MPP is a significant source of phenolic compounds that remain available within the dough matrix after processing. A similar pattern was observed for TFC, which increased from 22.46 mg QE/100 g DM in the control to 82.10 mg QE/100 g DM at 20% substitution, corresponding to an approximately 3.66-fold increase, highlighting the contribution of MPP to flavonoid enrichment in the dough system. Previous studies report that fortification of dough with functional ingredients can enhance phenolic content and antioxidant activity [63]. However, phenolic and flavonoid compounds contributed by the functional ingredient were not fully available in the dough matrix after processing. This limited availability is attributed to interactions with food matrix components, particularly proteins and starch, leading to the formation of complexes that reduce their extractability and detectability [64]. Partial replacement of WF with MPP improved the antioxidant profile of biscuit dough, significantly increasing its bioactive compound content, DPPH radical scavenging activity, and ferric reducing antioxidant power. This improvement highlights the potential of MPP as a value-added ingredient for developing enriched dough-based products.

**Figure 3:**
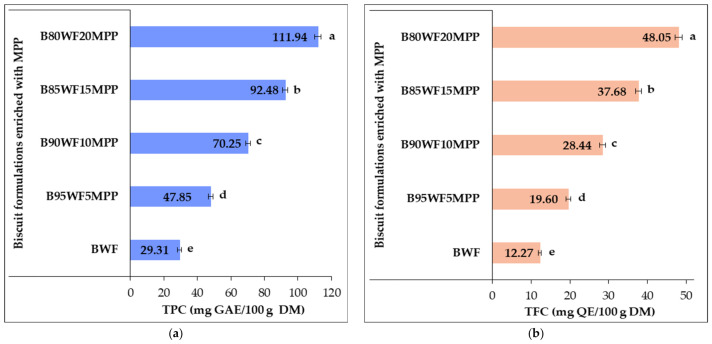
Total phenolic content (TPC) (**a**) and total flavonoid content (TFC) (**b**) of biscuit formulations across increasing levels of melon peel powder incorporation. BWF, B95WF5MPP, B90WF10MPP, B85WF15MPP, B80WF20MPP: biscuits with partial replacement of wheat flour by melon peel powder at 0, 5, 10, 15, and 20% (*w*/*w*). Values are expressed as mean ± SD from three independent replicates of each formulation. Values within bars bearing distinct letters (a–e) are significantly different (*p* < 0.05).

Figure 3 displays the total phenolic content (a) and total flavonoid content (b) in MPP-enriched biscuit formulations.

Incorporation of MPP into biscuit formulations led to a significant (*p* < 0.05) increase in TPC compared with the control, as shown in Figure 3a. The control sample exhibited a TPC of 29.31 mg GAE/100 g DM, whereas substitution of WF with MPP resulted in a progressive increase in this parameter. The highest content was recorded for B80WF20MPP (111.94 mg GAE/100 g DM), corresponding to an approximately 3.82-fold increase relative to the control. These results are consistent with previous studies showing that the incorporation of fruit- and vegetable-derived by-products into bakery products increases total phenolic content due to their intrinsic bioactive composition and antioxidant properties [22,65,66]. A similar threefold increase in TPC has been reported for MPP-fortified biscuits [24], supporting the enrichment observed in the present study. A wide range of phenolic compounds has been identified in melon peels [67], which further explains this upward trend. Similar tendencies have also been documented for biscuits enriched with pomegranate peel powder, where the substitution level positively influenced the TPC [68].

Substitution of WF with MPP resulted in a pronounced increase in TFC, as shown in Figure 3b. The control sample contained 12.27 mg QE/100 g DM, but the TFC increased progressively with MPP level, reaching 48.05 mg QE/100 g DM at 20% substitution, corresponding to a 3.92-fold increase. Similar increases in TFC have been reported in MPP-enriched biscuits [3], with a clear dependence on the substitution level. The presence of high flavonoid amounts in melon-related materials has also been confirmed in previous studies [69,70], supporting their contribution to the observed enrichment. The increase in flavonoids in bakery systems enriched with fruit and vegetable by-products is widely documented [32], and is mainly attributed to the intrinsic phytochemical composition of melon peels, where flavonoids represent one of the dominant phenolic classes [12]. Comparable trends have been reported for both TPC and TFC in cereal-based products enriched with powdered peel by-products, revealing their potential as a source of high-value-added compounds for food applications [6,70].

**Figure 4:**
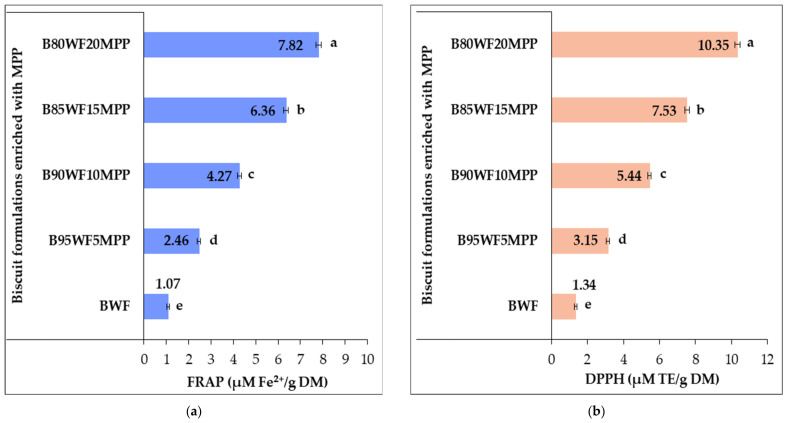
Ferric reducing antioxidant power (FRAP) (**a**) and DPPH radical scavenging activity (**b**) of biscuit prepared with different levels of melon peel powder. BWF, B95WF5MPP, B90WF10MPP, B85WF15MPP, B80WF20MPP: biscuits with partial replacement of wheat flour by melon peel powder at 0, 5, 10, 15, and 20% (*w*/*w*). Values are expressed as mean ± SD from three independent replicates of each formulation. Values within bars bearing distinct letters (a–e) are significantly different (*p* < 0.05).

Figure 4 illustrates the changes in ferric reducing antioxidant power (FRAP) and DPPH radical scavenging activity of biscuits with increasing level of MPP incorporation.

Both DPPH radical scavenging activity and ferric reducing antioxidant power (FRAP) showed a clear and statistically significant (*p* < 0.05) increase with increasing levels of MPP incorporation, confirming the contribution of MPP to the improved antioxidant properties of the biscuits. As shown in Figure 4a, the control biscuit (BWF) exhibited the lowest FRAP value (1.07 µM Fe^2+^/g DM). Progressive substitution of WF with MPP resulted in an increase in reducing power, reaching 7.82 µM Fe^2+^/g DM in B80WF20MPP, corresponding to an approximately 7.31-fold increase compared with the control sample. This improvement reflects the higher electron-donating capacity of phenolic compounds present in MPP, which reduce ferric ions (Fe^3+^) to ferrous ions (Fe^2+^), thereby enhancing the reducing power of the biscuit matrix. Similar increases in FRAP values in MPP-enriched bakery products have been reported by Silva et al. [24], who observed approximately a 5-fold increase relative to control formulations. Fruit peel powders are widely recognized as natural sources of antioxidants in bakery systems due to their high phenolic content [22,66]. As illustrated in Figure 4b, a comparable pattern was observed for DPPH radical scavenging activity. The control sample exhibited the lowest DPPH radical scavenging activity (1.34 µM TE/g DM), whereas the gradual incorporation of MPP resulted in a significant increase in scavenging activity across all biscuit formulations. The highest value was recorded for B80WF20MPP (10.35 µM TE/g DM), corresponding to an approximately 7.72-fold increase relative to the control. This improvement reflects enhanced radical scavenging potential induced by MPP addition. The activity is associated with the ability of phenolic and flavonoid compounds to donate hydrogen atoms or electrons, thereby neutralizing DPPH radicals and interrupting radical chain reactions. Increased DPPH activity in MPP-enriched biscuits has also been reported [3], while similar effects have been observed in biscuits fortified with fruit peel powders [68]. These effects are attributed to the high content of phenolics and flavonoids in melon peels, which enhance the antioxidant profile of biscuits [45] and support the potential of MPP as a value-added food ingredient.

### 3.6. Post-Baking Retention of Phytochemical Content, DPPH Radical Scaveging Activity and Ferric Reducing Antioxidant Power

**Figure 5:**
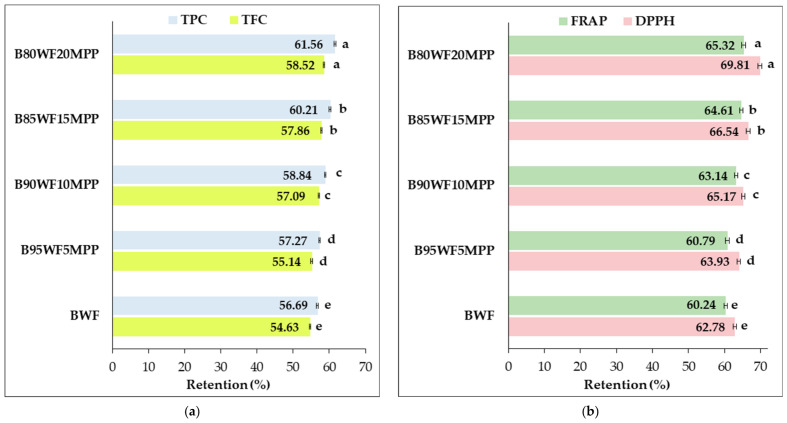
Post-baking retention (%) of total phenolic content and total flavonoid content (**a**), and ferric reducing antioxidant power (FRAP) and DPPH radical scavenging activity (**b**). BWF, B95WF5MPP, B90WF10MPP, B85WF15MPP, B80WF20MPP: biscuits with partial replacement of wheat flour by melon peel powder at 0, 5, 10, 15, and 20% (*w*/*w*). Values are expressed as mean ± SD from three independent replicates of each formulation. Values within bars bearing distinct letters (a–e) are significantly different (*p* < 0.05).

Baking may influence the retention of bioactive compounds due to heat-induced transformations, leading to differences between dough and final products. The post-baking retention of TPC, TFC, DPPH radical scavenging activity, and ferric reducing antioxidant power was expressed as the percentage of the values measured in MPP-enriched biscuit formulations relative to the corresponding dough values. The values for both dough and biscuit used to calculate post-baking retention were expressed on a dry-matter basis. Figure 5 presents the changes recorded in the post-baking retention of these parameters as a result of increasing levels of MPP incorporation.

The results indicate that although MPP incorporation significantly increased TPC, TFC, FRAP, and DPPH values at the dough stage, a decrease was observed after baking in all formulations, reflecting the impact of thermal processing on bioactive compound stability. This reduction is attributed to heat-induced degradation, oxidation reactions, and structural changes occurring during baking, which affect phenolic compounds and antioxidant molecules. Previous studies have shown that baking may simultaneously promote the degradation of thermolabile phenolics and the release of bound phenolic compounds from the food matrix [71,72,73]. The extent of these changes depends on phenolic profile, food matrix characteristics, formulation, and baking conditions [70]. Antioxidant properties may also be influenced by heat-induced modifications and changes in bioavailability [74,75].

Despite these losses, a substantial proportion of bioactive compounds was retained in the final biscuit products. Retention rates of TPC and TFC ranged from 56.69% to 61.56% and from 54.63% to 58.52%, respectively, indicating moderate thermal stability of phenolic and flavonoid compounds within the biscuit matrix.

Ferric reducing antioxidant power (FRAP) showed retention values ranging from 60.24% to 65.32%, while DPPH radical scavenging activity ranged from 62.78% to 69.81%, suggesting greater retention of FRAP and DPPH radical scavenging activity than of total phenolic and flavonoid contents during baking. The relatively high retention values observed may be attributed both to the release of bound phenolics during thermal processing [76] and to the formation of antioxidant-active Maillard reaction products. During baking, degradation of native antioxidants may be partially compensated by the generation of melanoidins and other Maillard-derived compounds with antioxidant properties [43,77].

Retention increased progressively with higher levels of MPP incorporation, suggesting that MPP contributes not only to the initial enrichment of bioactive compounds but also to their improved stability during baking. The presence of dietary fiber and phenolic–matrix interactions may have contributed to the partial protection of antioxidant compounds in the final products.

Differences between TPC/TFC and FRAP/DPPH radical scavenging activity retention further indicate that antioxidant properties is influenced not only by total phenolic content, but also by structural modifications and the formation of new antioxidant-active compounds during baking. Thermal processing may affect the stability and bioavailability of phenolic compounds, thereby modulating antioxidant properties independently of total phenolic levels [74,75]. These interactions highlight the complexity of thermal processing in multi-ingredient bakery systems and confirm that MPP-fortified biscuits retain a significant proportion of their bioactive potential after baking.

### 3.7. Characterization of WF, MPP, Composite Flours and Biscuit Formulations Using Fourier Transform Infrared Spectroscopy (FTIR)

**Figure 6:**
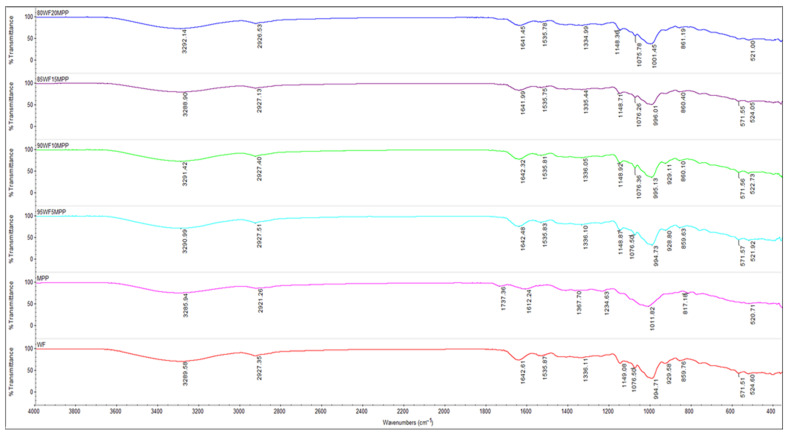
FTIR spectra of wheat flour, melon peel powder and composite flours in the spectral range 4000–400 cm^−1^, 32 scans at 4 cm^−1^ resolution. WF: wheat flour; MPP: melon peel powder; 95WF5MPP, 90WF10MPP, 85WF15MPP, 80WF20MPP: composite flours with partial replacement of WF by MPP at 0, 5, 10, 15, and 20% (*w*/*w*).

The FTIR spectra of WF, MPP, and WF–MPP composite flours, presented in Figure 6, exhibited similar spectral features characteristic of starch- and polysaccharide-rich systems, with no evidence of new functional groups following MPP substitution, while the observed changes were mainly related to variations in band intensity and, to a lesser extent, slight shifts in band position, suggesting interactions among starch, gluten proteins, dietary fiber, and pectic compounds derived from MPP.

A broad absorption band was observed in all samples within the 3285–3292 cm^−1^ region and was attributed to O–H stretching vibrations of hydroxyl groups involved in hydrogen-bonding interactions, originating from starch, cellulose, hemicelluloses, pectins, and bound water [78,79]. In the MPP spectrum, this band appeared at 3285 cm^−1^, consistent with O–H absorption bands previously reported for melon peel and pectin extracted from melon peel [79,80]. In WF, the band was observed at 3289 cm^−1^, while in the WF–MPP composite flours it ranged from 3288 to 3292 cm^−1^, without a progressive shift with increasing MPP substitution, in agreement with Naseem et al. [81]. The slight variations in intensity and position are consistent with the additional contribution of hydroxyl groups from the fibrous and pectic fractions of MPP, without indicating major structural modifications in the system. These observations suggest that the hydroxyl-rich fiber and pectic constituents of MPP participate in the existing hydrogen-bonding network of the flour matrix, leading to a reorganization of intermolecular hydrogen-bonding interactions rather than the formation of new covalent linkages.

A medium-intensity band in the 2921–2927 cm^−1^ region, assigned to C–H stretching vibrations of methylene (–CH_2_) and methyl (–CH_3_) groups present in polysaccharides and other organic matrix constituents [79,82], was detected at 2921 cm^−1^ in MPP and at 2927 cm^−1^ in WF. In the composite flours, the band position remained within the 2926–2927 cm^−1^ range, close to that of WF, suggesting that MPP incorporation did not substantially alter the structural environment of aliphatic C–H groups.

A distinct band at 1737 cm^−1^ was observed exclusively in the MPP spectrum and was attributed to C=O stretching vibrations of esterified carbonyl groups associated with the pectic fraction and other esterified components of the plant cell wall [79,83,84]. This band was not clearly detected in the composite flour spectra, which can be attributed to the low concentration of MPP and overlap with the more intense absorption bands of the starch–protein matrix. Bands observed in the 1641–1642 cm^−1^ region were attributed mainly to bending vibrations of bound water and, to a lesser extent, to the Amide I band (~1635–1655 cm^−1^) associated with gluten proteins. Minor shifts within this region in the composite flours may reflect a reorganization of hydrogen-bonding interactions between starch, gluten proteins, and dietary fiber.

In the MPP spectrum, the corresponding band appeared at 1612 cm^−1^ and was associated with carboxylate (COO^−^) groups from pectins and other acidic polysaccharides in the plant cell wall [79,83]. The Amide II band (~1536 cm^−1^), related to N–H bending and C–N stretching vibrations of proteins [83], remained essentially unchanged in all composite flour samples, suggesting no major modifications in the protein fraction at the tested substitution levels. In the fingerprint region, the band at 1335–1336 cm^−1^ in WF and composite flours and at 1367 cm^−1^ in MPP, attributed to C–H deformation and glycosidic ring vibrations [78], reflects compositional differences in polysaccharide fractions, particularly the presence of pectic and hemicellulosic components in MPP.

The band at ~1234 cm^−1^, observed only in the MPP spectrum and assigned to C–O stretching vibrations of ester groups in pectins [83], was not clearly detected in WF or composite flours, which can be attributed to overlapping with stronger absorption bands of the starch-rich matrix. The bands at 1148–1149 cm^−1^ and ~1075 cm^−1^, assigned to C–C and C–O stretching vibrations of polysaccharides [85], remained essentially unchanged in all composite flours, indicating that the starch-rich matrix was largely preserved following MPP incorporation. Similarly, the bands in the 995–1011 cm^−1^ region, associated with C–O–C and C–O stretching vibrations of glucosidic structures characteristic of starch [85], did not show major spectral changes with increasing MPP level, suggesting preservation of the main polysaccharide structure. The observed intensity variations in this region are consistent with the contribution of fibrous and pectic compounds from MPP [86].

The band at approximately 817 cm^−1^ in the MPP spectrum may be associated with skeletal vibrations of polysaccharide structures characteristic of the plant cell wall [78]. In the low-wavenumber fingerprint region, bands at ~571 cm^−1^ and 521–524 cm^−1^ were identified in WF and all composite flours and were attributed to skeletal bending vibrations of carbohydrate rings and C–O–H deformation modes in polysaccharides [78]. The absence of the ~571 cm^−1^ band in the MPP spectrum, where only the ~520 cm^−1^ band was observed, reflects differences in polysaccharide composition between melon peel and wheat starch. Moreover, the slightly increased intensity of these bands in the composite flours is consistent with the contribution of MPP components rich in dietary fiber and pectins.

The absence of new absorption bands in the FTIR spectra indicates that MPP incorporation did not induce the formation of new covalent bonds within the flour matrix. Instead, the slight variations in band position and intensity suggest subtle changes in the hydrogen-bonding environment associated with the hydroxyl-rich fiber and pectic constituents of MPP. The shift in the O–H stretching band from 3289 cm^−1^ in WF to 3292 cm^−1^ the 80WF20MPP blend reflects a minor restructuring of the intermolecular hydrogen bonds, indicating that the pectic hydroxyl groups interact with the flour components through these weak physical pathways without forming stable covalent networks.

The minimal changes observed in the Amide I and Amide II regions further indicate that the gluten protein structure remained largely preserved, suggesting that any interactions between pectic compounds and gluten proteins were predominantly non-covalent.

**Figure 7:**
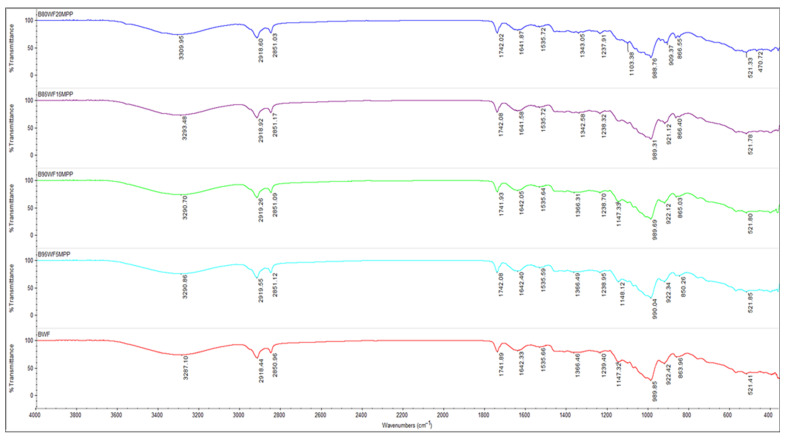
FTIR spectra of biscuit samples based on wheat flour enriched with melon peel powder in the spectral range 4000–400 cm^−1^, 32 scans at 4 cm^−1^ resolution. BWF, B95WF5MPP, B90WF10MPP, B85WF15MPP, B80WF20MPP: biscuits with partial replacement of wheat flour by melon peel powder at 0, 5, 10, 15, and 20% (*w*/*w*).

The FTIR characteristics of the flour samples provide a reference for interpreting the spectra of the baked biscuits. Accordingly, the FTIR spectra of the biscuit samples (Figure 7) revealed structural modifications relative to the corresponding flour formulations, reflecting physicochemical transformations induced during baking, including starch gelatinization and molecular reorganization, gluten protein denaturation, lipid-related contributions, and interactions among food matrix components.

All formulations exhibited similar spectral profiles with no evidence of new functional groups, while the observed changes were mainly related to variations in band intensity and position of bands already present in the system. These findings indicate that baking primarily modified the molecular organization of the existing matrix components rather than inducing the formation of new chemical structures.

The broad band in the 3287–3309 cm^−1^ region, attributed to O–H stretching vibrations of hydroxyl groups from starch, dietary fiber, pectins, and bound water, shifted progressively toward higher wavenumbers with increasing MPP substitution level, suggesting a reorganization of the hydrogen-bonding network associated with the incorporation of dietary fibers and pectins from MPP into the gelatinized starch matrix [78,87].

All biscuit spectra exhibited a characteristic doublet at approximately 2918–2919 cm^−1^ and 2850–2851 cm^−1^, corresponding to asymmetric and symmetric C–H stretching vibrations of methylene groups (–CH_2_–) in fatty acid chains [88]. This doublet was absent in the composite flour spectra, where a single band at ~2927 cm^−1^ was observed, assigned to C–H stretching vibrations of glucosidic units in starch, indicating a reduced lipid contribution in the flour matrix compared with the baked products [85].

A prominent band at approximately 1741–1742 cm^−1^, absent in the composite flour spectra, was attributed to C=O stretching vibrations of esterified carbonyl groups mainly originating from lipids introduced via the biscuit ingredients, with a possible minor contribution from ester groups in the pectic fraction of MPP [87], and represents a key spectral feature distinguishing biscuit matrices from composite flours.

The Amide I (~1641–1642 cm^−1^) and Amide II (~1535 cm^−1^) bands, recognized as sensitive markers of protein conformation in FTIR analysis of food products [89], were retained in all biscuit spectra, with variations in relative intensity associated with thermal denaturation and reorganization of the secondary structure of gluten proteins during baking. The preservation of the Amide I and Amide II bands suggests that, despite the physicochemical changes induced by baking, interactions between MPP-derived pectic polysaccharides and gluten proteins did not result in major alterations of the overall protein secondary structure, suggesting that these interactions remained predominantly non-covalent.

The band in the 1342–1366 cm^−1^ region, attributed to C–H deformation and glycosidic ring vibrations [78], was present in all biscuit samples, with a shift toward lower wavenumbers at higher substitution levels (B85WF15MPP and B80WF20MPP), consistent with modifications in the molecular environment of carbohydrate structures induced by increasing levels of fibrous and pectic components from MPP.

The progressive intensification of bands at ~1237–1239 cm^−1^ and ~1147–1148 cm^−1^, attributed to C–O and C–O–C stretching vibrations characteristic of pectins and dietary fiber [85], was more pronounced in biscuits than in the corresponding composite flours, confirming the retention and integration of pectic and fibrous components from MPP into the product matrix after baking.

The modifications observed in the 988–1150 cm^−1^ region, characteristic of carbohydrate structures in wheat starch [85], indicate a reduction in the native crystalline order of starch granules and are consistent with starch gelatinization during baking, as well as interactions between MPP fibers and the gelatinized starch matrix [87]. The reduction in intensity and broadening of the peaks within this region confirms the transition of the carbohydrate matrix toward an amorphous state during thermal processing. The retention of this broad profile across all MPP-substituted biscuits indicates that the non-starch polysaccharides restrict the regular reassociation of starch chains during cooling, thereby limiting the extent of retrogradation in the final matrix.

The band in the 850–866 cm^−1^ region, associated with C–OH and CH_2_ deformation modes and glycosidic linkage vibrations in polysaccharide structures [85], was identified in all biscuit samples without major spectral changes as a function of MPP level, confirming the structural stability of the carbohydrate matrix following baking.

The band at ~521 cm^−1^, attributed to skeletal bending vibrations of carbohydrate rings [77], was present in all biscuit samples, with minor intensity variations consistent with maintained interactions between polysaccharides from MPP and the gelatinized starch matrix after baking.

Comparative analysis of the composite flour spectra with those of the corresponding biscuit samples clearly demonstrates structural modifications induced by the baking process, including reorganization of the hydrogen-bonding network associated with starch gelatinization, the appearance of signals characteristic of the lipid fraction, and changes in the fingerprint region. Partial substitution of WF with MPP up to 20% did not fundamentally alter the molecular structure of the biscuit matrix, but rather promoted the incorporation and retention of pectic and fibrous components from melon peel, as evidenced by the progressive intensification of their characteristic bands with increasing substitution level. The retention of bands associated with pectins and polysaccharides from MPP after thermal treatment confirms their integration into the final product matrix and supports the potential of MPP as a value-added ingredient for the development of biscuits enriched in dietary fiber and bioactive compounds.

### 3.8. Wide-Angle X-Ray Scattering (WAXS) Analysis of WF, MPP, Composite Flours and Biscuit Formulations

**Figure 8:**
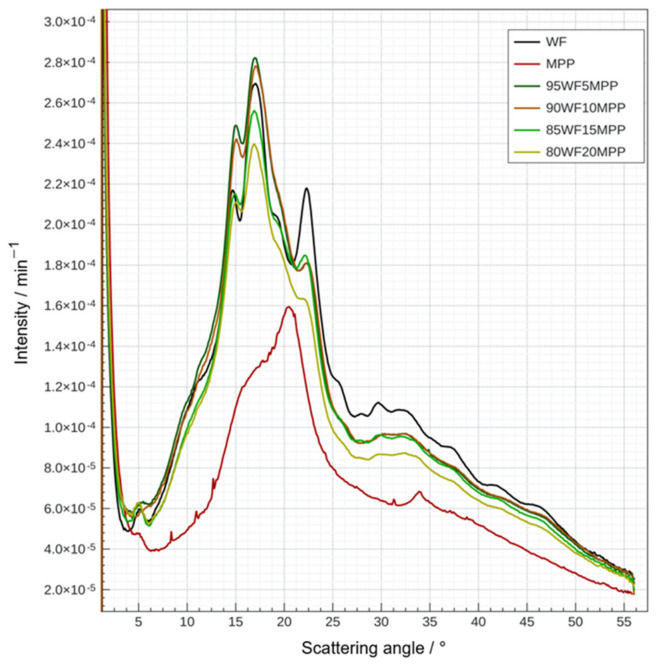
Wide-Angle X-ray Scattering (WAXS) analysis of wheat flour, melon peel powder and composite flours. WF: wheat flour; MPP: melon peel powder; 95WF5MPP, 90WF10MPP, 85WF15MPP, 80WF20MPP: composite flours with partial replacement of WF by MPP at 0, 5, 10, 15, and 20% (*w*/*w*).

WAXS analysis was performed to evaluate structural changes in the starch matrix of WF in the presence of MPP, both in composite flours and the resulting biscuit formulations, providing valuable insight into the crystalline organization of starch granules and the associated protein–starch interactions in WF affected by MPP incorporation. The WAXS profiles of WF, MPP and WF-MPP composite flours are displayed in Figure 8.

The changes observed in the intensity and width of diffraction maxima suggest that MPP incorporation influenced the structural organization of the starch phase and the balance between crystalline and amorphous regions of the system. The WAXS profile of WF exhibited the typical features of A-type starch, with a secondary maximum at a scattering angle (2θ) of approximately 15°, a pronounced main peak at 2θ ≈ 17–18°, and a visible shoulder at 2θ ≈ 21–23°, corresponding to the monoclinic crystalline structure characteristic of cereal starch. This diffraction pattern is associated with the ordered arrangement of amylopectin double helices forming the crystalline regions of starch granules [90,91]. MPP exhibited a markedly different WAXS profile compared with WF (Figure 8), characterized by considerably lower overall intensity, broad and poorly defined peaks, and the absence of sharp secondary reflections typical of crystalline starch. This pattern is indicative of a predominantly amorphous structure, characteristic of plant-based materials rich in dietary fiber and cell wall components, which exhibit limited long-range crystalline order [36]. A low-intensity signal at small scattering angles (2θ ≈ 6°) in the MPP profile may be associated with partially ordered structures of pectin or hemicellulose origin [87]. The WF–MPP composite flours retained the characteristic diffraction peaks of WF at scattering angles 2θ ≈ 15° and 2θ ≈ 17–18°, confirming the preservation of A-type crystallinity specific to wheat starch regardless of MPP level. This indicates that MPP addition does not induce a polymorphic transition but rather modifies the degree of ordering within the same crystalline system. The total stability of these diffraction peak positions and the absence of any new diffraction maxima provide direct spectral evidence that the native starch crystal structure remains intact, indicating that no new crystalline phases or covalent networks are formed between the WF matrix and MPP.

With increasing MPP content, a slight variation in the intensity of the main peak at 2θ ≈ 17–18° was observed, together with modification of the secondary peak at 2θ ≈ 15° and of reflections in the 2θ = 22–36° range. This fluctuation in diffraction intensity suggests an increased contribution of amorphous regions due to the incorporation of the fiber- and pectin-rich MPP fraction, while the preservation of the A-type diffraction pattern indicates that the crystalline polymorph of wheat starch remained unchanged.

Because no new diffraction reflections or modifications in the 2θ peak positions are registered, these intensity variations reflect a non-covalent structural rearrangement. This behavior is governed primarily by physical dilution and steric coexistence between the amorphous MPP non-starch polysaccharides and the crystalline starch matrix.

The incorporation of MPP-derived fiber and pectic components may restrict the molecular mobility of starch chains, limiting their ability to form highly ordered arrangements and contributing to the increased amorphous character of the matrix.

These changes are consistent with dilution of the crystalline phase due to the incorporation of a predominantly amorphous fraction from MPP, leading to reduced relative ordering of starch crystalline regions [87,91]. This behavior agrees with previous reports on starch systems enriched with dietary fiber and fruit-derived powders [36,87].

**Figure 9:**
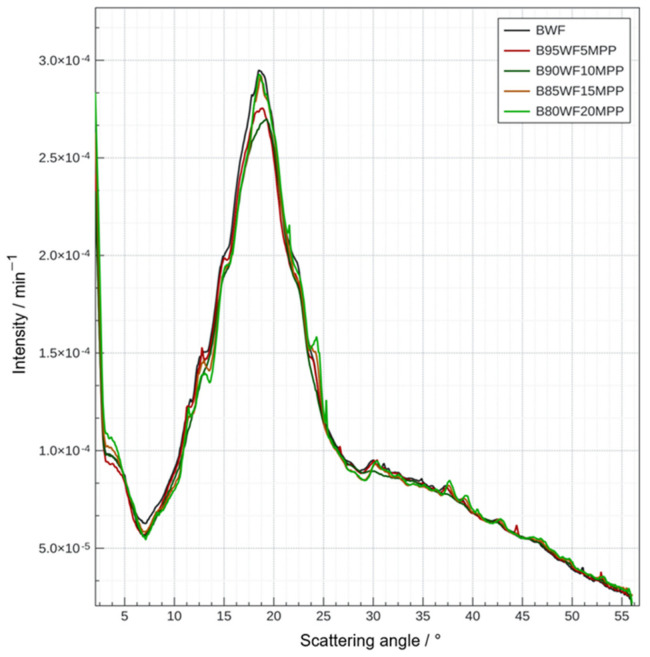
Wide-Angle X-ray Scattering (WAXS) analysis of biscuit samples based on wheat flour enriched with melon peel powder. BWF, B95WF5MPP, B90WF10MPP, B85WF15MPP, B80WF20MPP: biscuits with partial replacement of wheat flour by melon peel powder at 0, 5, 10, 15, and 20% (*w*/*w*).

The WAXS profiles of biscuit samples, Figure 9, differ considerably from those of the corresponding native flours and show high uniformity across formulations, with nearly overlapping curves over the entire angular range.

The main diffraction maximum in baked biscuit samples appears at a scattering angle of 2θ ≈ 19–20°, showing a broader profile compared with raw flours. In contrast, the secondary peak at 2θ ≈ 15° and the reflections in the 2θ = 22–36° range observed in WF are strongly attenuated or absent after baking. These structural changes result from baking at high temperature, which induces extensive starch gelatinization and a substantial loss of native A-type crystallinity. During cooling, partial retrogradation occurs, mainly involving amylose, leading to the formation of a less ordered crystalline structure characterized by broader and less intense diffraction maxima [92,93]. The presence of non-starch polysaccharides from MPP may interfere with starch chain association during cooling, reducing the extent of ordered rearrangements associated with retrogradation. This is experimentally demonstrated by the nearly overlapping curves in Figure 9, confirming that thermal processing is the dominant factor governing the final structure fingerprint of the biscuits. Such an interpretation aligns with the slightly more pronounced low-angle scattering observed in biscuits containing higher MPP levels, suggesting that the amorphous fraction introduced by MPP remained after baking.

The strong similarity of WAXS profiles across biscuit formulations indicates that thermal processing is the dominant factor governing the final structure, largely masking differences introduced by MPP at the WF level. Nevertheless, a more detailed comparison shows that biscuits with higher MPP levels (B85WF15MPP and B80WF20MPP) exhibit a slightly more pronounced signal in the amorphous region (2θ < 12°) compared with BWF. This suggests that part of the amorphous fraction introduced by MPP persists after baking. This behavior may be associated with dietary fiber, pectins, and other non-starch components from MPP, which contribute to increased amorphous character and modify the structural organization of the starch matrix [36,87]. MPP components may also modify water availability within the matrix, influencing starch chain mobility and the extent of molecular rearrangement during baking and subsequent cooling.

WAXS analysis revealed that partial substitution of WF with MPP does not alter the crystalline type of starch but induces a gradual decrease in the relative ordering of crystalline regions. In contrast, the baking process leads to a reorganization of the starch structure and results in highly similar WAXS profiles across biscuit formulations, with a slightly greater contribution of the amorphous fraction in samples with higher MPP levels.

## 4. Conclusions

This study demonstrated the potential of melon peel powder (MPP), characterized by high phytochemical content, DPPH radical scavenging activity and ferric reducing antioxidant power, as a value-added ingredient for biscuit formulations. Incorporation of MPP at levels up to 20% successfully combined nutritional enrichment, through increased dietary fiber and ash contents and reduced available carbohydrates and energy value, with enhanced phytochemical composition and antioxidant properties. From a technological perspective, MPP influenced baking behavior and colour development, increasing baking yield and spread ratio while promoting darker yellow-brown tones of the biscuits. Despite thermal processing, substantial retention of bioactive compounds and antioxidant properties was maintained (55–70%) at the highest MPP level, indicating moderate thermal stability of bioactive constituents and a possible contribution of heat-induced antioxidant compounds, while the structural integrity of the biscuit matrix was preserved. FTIR and WAXS data confirmed that the WF-MPP composite flours maintain structural compatibility within the biscuit matrix, as shown by the absence of new functional groups, the absence of polymorphic shifts, and the preservation of the A-type starch crystalline structure without phase separation. Additionally, the baking process led to starch gelatinization and the structural incorporation of fibrous and pectic components, resulting in a slightly higher amorphous fraction. These findings provide a comprehensive understanding of how fruit by-products interact with cereal-based systems and influence bakery product characteristics. The results demonstrate that incorporation of MPP up to 20% represents a promising strategy for the valorisation of melon processing by-products and for the development of bakery products with improved nutritional and antioxidant properties. Beyond these effects, this study provides experimental evidence linking compositional, phytochemical, technological, and structural responses within a single system. Future research should focus on sensory evaluation, texture analysis, long-term storage stability, and profiling of phenolic compounds to better understand their stability during processing and baking. Further studies may also investigate the behaviour of MPP-enriched dough systems under different processing conditions, including rheological characterization, to refine structure–function relationships in composite flour systems.

## Figures and Tables

**Table 1:** Application of melon by-products in bakery products.

Melon Variety/ Ingredient	Bakery Product	Substitution Level	Relevant Findings	Limitations	Reference
*Cucumis melo* var. Fonzy/Canary melon peel powder; *Citrullus lanatus* var. Augusta/watermelon peel powder; *Cucumis melo* var. Cantaloupe/muskmelon peel powder	Biscuits	5 and 10% WF replacement	Increased moisture, ash, fat, fiber, minerals, phenolics, flavonoids, and antioxidant activity. Maintained sensory acceptability at 5% substitution, whereas 10% further improved nutritional and antioxidant properties but reduced sensory scores.	No storage stability assessment. No instrumental texture analysis.	[3]
*Cucumis melo* L./melon peel flour	Cupcakes	4.25, 8.5, 12.75 and 17% WF replacement	Maintained sensory acceptability up to 12.75% substitution. Increased ash and dietary fiber improving the nutritional profile.	No storage stability assessment. No instrumental texture analysis.	[21]
*Cucumis melo* L./melon peel flour	Cookies	15% WF replacement	Enhanced antioxidant activity and total phenolic content.	No sensory evaluation.	[22]
*Cucumis melo* L./sweet melon peel flour and seed flour	Cookies	15% WF replacement with melon peel flour; 15% WF replacement with melon seed flour	Improved amino acid profile. Maintained acceptable sensory quality.	No storage stability assessment.	[23]
*Cucumis melo* L./melon peel flour	Biscuits; Muffins	50% WF replacement in biscuits; 100% WF replacement in muffins	Increased dietary fiber, total phenolic content, and antioxidant activity. Maintained satisfactory sensory properties.	Single substitution level evaluated. No storage stability assessment.	[24]
*Cucumis melo* L./Sharlyn melon peel powder; *Citrullus lanatus* L./Watermelon rind powder	Cakes	2.5, 5 and 7.5% WF substitution; 5, 10 and 15% fat substitution	Improved volume and specific volume. Maintained acceptable sensory quality up to 5% flour replacement. Reduced staling and lipid oxidation during storage and maintained acceptable sensory quality up to 10% fat replacement.	No instrumental texture analysis.	[25]
*Cucumis melo* var. *inodorus* cv. Manis Terengganu 1/melon peel powder	Bread	3, 6, 9 and 12% WF replacement	Increased crude fiber and moisture content. Decreased protein and carbohydrate contents, and loaf volume. Best sensory acceptability at 3% substitution.	No storage stability assessment.	[26]
*Cucumis melo* L./melon peel flour	Muffins	11% WF replacement	Increased phenolic content, antioxidant activity, and dietary fiber content.	No storage stability assessment.	[27]
*Cucumis melo* L./melon peel flour	Muffins	10 and 20% rice flour replacement	Increased fiber, ash, and protein contents. Changed colour. Maintained sensory acceptability at 10% substitution, whereas 20% reduced sensory acceptability and technological quality.	No storage stability assessment.	[28]
*Cucumis melo* L./Maazoun melon peel powder	Cakes	10%, 20% and 30% WF replacement	Improved fiber content, texture, and sensory quality Sensory quality maintained at 10%. Darker color and lower acceptability at higher levels.	No storage stability assessment.	[29]
*Cucumis melo* L./melon seed flour	Cakes	40 and 60% WF replacement	Improved protein, fiber, ash, and lipid content. High replacement levels may alter product quality and acceptability.	No storage stability assessment.	[30]
*Cucumis melo* L. var. reticulatus/Cantaloupe melon seed flour	Cakes	40 and 60% wheat flour replacement	Improved protein, fiber, ash, and lipid content. High replacement levels may alter product quality and acceptability.	No storage stability assessment. No instrumental texture analysis.	[31]
*Cucumis melo* L./melon peel flour; melon seed flour	Biscuits	2.5% and 5% WF replacement with melon peel flour, alone or combined with 1.25% and 2.5% melon seed flour	Melon peel flour: Increased cookie spread, softened texture, and maintained acceptable sensory quality up to 5% substitution. Melon peel flour + melon seed flour: Improved nutritional value while maintaining good sensory acceptability; the combination of 5% peel flour and 2.5% seed flour produced nutritionally enriched cookies.	No storage stability assessment.	[32]

WF: wheat flour.

**Table 2:** Ingredients for biscuit formulation enriched with melon peel powder.

Ingredients	BWF	B95WF5MPP	B90WF10MPP	B85WF15MPP	B80WF20MPP
WF (g)	200	190	180	170	160
MPP (g)	-	10	20	30	40
Sugar (g)	68	68	68	68	68
Butter (g)	100	100	100	100	100
Eggs (g)	50	50	50	50	50
Baking powder (g)	3	3	3	3	3
Salt (g)	1	1	1	1	1

WF: wheat flour; MPP: melon peel powder. BWF, B95WF5MPP, B90WF10MPP, B85WF15MPP, and B80WF20MPP: biscuits with partial replacement of WF by MPP at 0, 5, 10, 15, and 20% (*w*/*w*).

**Table 3:** Proximate composition and energy value of wheat flour and melon peel powder.

Sample	Component (%)						Energy Value (kcal/100 g)
	Moisture	Protein	Fat	Ash	Total Dietary Fiber	Available CRB	
WF	12.89 ± 0.03 ^a^	11.48 ± 0.05 ^a^	1.28 ± 0.03 ^b^	0.34 ± 0.01 ^b^	0.46 ± 0.03 ^b^	73.55 ± 0.05 ^a^	351.64 ± 0.06 ^a^
MPP	4.97 ± 0.02 ^b^	10.09 ± 0.04 ^b^	1.66 ± 0.04 ^a^	9.71 ± 0.03 ^a^	28.39 ± 0.04 ^a^	45.18 ± 0.04 ^b^	236.02 ± 0.04 ^b^

CRB: carbohydrates; WF: wheat flour; MPP: melon peel powder. Data are expressed as mean ± SD of three independent analyses. Values within the same column bearing distinct superscript letters (a, b) are significantly different (*p* < 0.05).

**Table 4:** Proximate composition and energy value of MPP-enriched biscuit formulations.

Component (%)	Samples				
	BWF	B95WF5MPP	B90WF10MPP	B85WF15MPP	B80WF20MPP
Moisture	5.08 ± 0.02 ^e^	5.41 ± 0.03 ^d^	5.68 ± 0.03 ^c^	5.97 ± 0.02 ^b^	6.33 ± 0.04 ^a^
Protein	8.07 ± 0.03 ^a^	8.05 ± 0.02 ^a^	8.02 ± 0.03 ^a^	7.98 ± 0.02 ^a^	7.96 ± 0.03 ^a^
Fat	23.29 ± 0.02 ^d^	23.36 ± 0.02 ^c^	23.44 ± 0.03 ^b^	23.49 ± 0.04 ^b^	23.56 ± 0.02 ^a^
Ash	1.03 ± 0.02 ^e^	1.28 ± 0.03 ^d^	1.51 ± 0.04 ^c^	1.77 ± 0.02 ^b^	2.01 ± 0.04 ^a^
Total Dietary Fiber	0.25 ± 0.03 ^e^	0.99 ± 0.04 ^d^	1.73 ± 0.03 ^c^	2.41 ± 0.05 ^b^	3.19 ± 0.06 ^a^
Available CRB	62.28 ± 0.04 ^a^	60.91 ± 0.02 ^b^	59.62 ± 0.04 ^c^	58.37 ± 0.03 ^d^	56.93 ± 0.03 ^e^
Energy Value (kcal/100 g)	491.01 ± 0.04 ^a^	486.08 ± 0.05 ^d^	481.52 ± 0.05 ^d^	476.85 ± 0.04 ^e^	471.68 ± 0.03 ^e^

CRB: carbohydrates; BWF, B95WF5MPP, B90WF10MPP, B85WF15MPP, B80WF20MPP: biscuits with partial replacement of WF by MPP at 0, 5, 10, 15, and 20% (*w*/*w*). Values are expressed as mean ± SD from three independent replicates of each formulation. Values within the same row bearing distinct superscript letters (a–e) are significantly different (*p* < 0.05).

**Table 5:** Color properties of biscuit formulations enriched with melon peel powder.

Color Parameters	Samples				
	BWF	B95WF5MPP	B90WF10MPP	B85WF15MPP	B80WF20MPP
L*	79.97 ± 0.04 ^a^	79.15 ± 0.03 ^b^	77.04 ± 0.04 ^c^	76.14 ± 0.04 ^d^	74.85 ± 0.05 ^e^
a*	4.21 ± 0.02 ^e^	4.60 ± 0.02 ^d^	5.11 ± 0.01 ^c^	5.60 ± 0.02 ^b^	5.89 ± 0.02 ^a^
b*	26.08 ± 0.03 ^e^	27.60 ± 0.05 ^d^	28.63 ± 0.04 ^c^	30.06 ± 0.02 ^b^	31.11 ± 0.02 ^a^
C	26.41 ± 0.03 ^e^	27.98 ± 0.05 ^d^	29.08 ± 0.04 ^c^	30.58 ± 0.02 ^b^	31.66 ± 0.02 ^a^
TCI	84.22 ± 0.03 ^a^	83.95 ± 0.02 ^b^	82.34 ± 0.05 ^c^	82.05 ± 0.03 ^d^	81.27 ± 0.04 ^e^
h°	80.83 ± 0.03 ^a^	80.54 ± 0.04 ^b^	79.87 ± 0.04 ^c^	79.45 ± 0.03 ^d^	79.28 ± 0.03 ^e^
∆E	-	1.77 ± 0.02 ^d^	3.99 ± 0.05 ^c^	5.70 ± 0.04 ^b^	7.37 ± 0.04 ^a^
BI	42.52 ± 0.09 ^e^	46.24 ± 0.11 ^d^	50.33 ± 0.04 ^c^	54.47 ± 0.05 ^b^	58.19 ± 0.08 ^a^

L*: lightness; a*: red–green coordinate; b*: yellow–blue coordinate; ΔE: total color difference between melon peel powder–enriched samples and the control; C: chroma; h°: hue angle; TCI: total color intensity; BI: browning index. BWF, B95WF5MPP, B90WF10MPP, B85WF15MPP, B80WF20MPP: biscuits with partial replacement of wheat flour by melon peel powder at 0, 5, 10, 15, and 20% (*w*/*w*). Values are expressed as mean ± SD from three independent replicates of each formulation. Values within the same row bearing distinct superscript letters (a–e) are significantly different (*p* < 0.05).

**Table 6:** Baking performance and physical characteristics of MPP-enriched biscuit formulations.

Sample	Weight (g)	Weight Loss (g)	Baking Yield (%)	Diameter (mm)	Thickness (mm)	Spread Ratio	Spread Factor (%)
BWF	362.78 ± 0.12 ^e^	59.22 ± 0.11 ^a^	85.97 ± 0.06 ^e^	58.78 ± 0.04 ^a^	11.07 ± 0.05 ^a^	5.31 ± 0.01 ^e^	100.00
B95WF5MPP	364.04 ± 0.10 ^d^	57.96 ± 0.07 ^b^	86.27 ± 0.08 ^d^	58.31 ± 0.03 ^b^	10.71 ± 0.04 ^b^	5.44 ± 0.02 ^d^	102.52
B90WF10MPP	365.09 ± 0.09 ^c^	56.91 ± 0.05 ^c^	86.51 ± 0.10 ^c^	57.53 ± 0.05 ^c^	10.35 ± 0.03 ^c^	5.56 ± 0.03 ^c^	104.66
B85WF15MPP	366.21 ± 0.11 ^b^	55.19 ± 0.08 ^d^	86.78 ± 0.06 ^b^	56.61 ± 0.07 ^d^	9.94 ± 0.04 ^d^	5.70 ± 0.01 ^b^	107.24
B80WF20MPP	367.62 ± 0.14 ^a^	54.38 ± 0.10 ^e^	87.11 ± 0.09 ^a^	56.12 ± 0.04 ^e^	9.56 ± 0.02 ^e^	5.87 ± 0.03 ^a^	110.54

BWF, B95WF5MPP, B90WF10MPP, B85WF15MPP, B80WF20MPP: biscuits with partial replacement of wheat flour by melon peel powder at 0, 5, 10, 15, and 20% (*w*/*w*). Values are expressed as mean ± SD from three independent replicates of each formulation. Values within the same column bearing distinct superscript letters (a–e) are significantly different (*p* < 0.05).

**Table 7:** Phytochemical content, DPPH radical scavenging activity, and ferric reducing antioxidant power (FRAP) of wheat flour and melon peel powder.

Sample	TPC (mg GAE/100 g DM)	TFC (mg QE/100 g DM)	FRAP (µM Fe^2+^/g DM)	DPPH (µM TE/g DM)
WF	99.41 ± 0.57 ^b^	41.94 ± 1.06 ^b^	2.74 ± 0.05 ^b^	3.28 ± 0.06 ^b^
MPP	1531.76 ± 1.41 ^a^	681.42 ± 1.29 ^a^	104.37 ± 1.19 ^a^	125.01 ± 1.27 ^a^

WF: wheat flour; MPP: melon eel powder; Data are expressed as mean ± SD of three independent analyses. Values within the same column bearing distinct superscript letters (a, b) are significantly different (*p* < 0.05).

**Table 8:** Phytochemical content, DPPH radical scavenging activity, and ferric reducing antioxidant power (FRAP) of biscuit dough formulations.

Sample	TPC (mg GAE/100 g DM)	TFC (mg QE/100 g DM)	FRAP (µM Fe^2+^/g DM)	DPPH (µM TE/g DM)
DWF	51.71 ± 0.61 ^e^	22.46 ± 0.34 ^e^	1.78 ± 0.04 ^e^	2.14 ± 0.06 ^e^
D95WF5MPP	83.55 ±1.07 ^d^	35.54 ± 0.49 ^d^	4.04 ± 0.06 ^d^	4.93 ± 0.098 ^d^
D90WF10MPP	119.40 ± 1.29 ^c^	49.82 ± 0.58 ^c^	6.77 ± 0.05 ^c^	8.35 ± 0.11 ^c^
D85WF15MPP	153.60 ± 1.36 ^b^	65.12 ± 0.65 ^b^	9.84 ± 0.09 ^b^	11.31 ± 0.14 ^b^
D80WF20MPP	181.84 ± 1.53 ^a^	82.10 ± 1.14 ^a^	11.97 ± 0.10 ^a^	14.83 ± 0.19 ^a^

DWF, D95WF5MPP, D90WF10MPP, D85WF15MPP, D80WF20MPP: biscuit dough formulations with partial replacement of wheat flour by melon peel powder at 0, 5, 10, 15, and 20% (*w*/*w*). Values are expressed as mean ± SD from three independent replicates of each formulation. Values within the same column bearing distinct superscript letters (a–e) are significantly different (*p* < 0.05).

## Data Availability

The original contributions presented in this study are included in the article. Further inquiries can be directed to the corresponding author.
